# *Periplaneta americana L.* extract exerts neuroprotective effects by inhibiting endoplasmic reticulum stress via AKT-dependent pathway in experimental models of Parkinson’s disease

**DOI:** 10.1186/s13020-024-01029-2

**Published:** 2024-11-13

**Authors:** Ting Cao, Xue-lian Wang, Jiang-yan Rao, Hui-feng Zhu, Hong-yi Qi, Zhen Tian

**Affiliations:** College of Pharmaceutical Sciences, Southwest University, No.1 Tiansheng Road, Beibei District, Chongqing, 400715 China

**Keywords:** Parkinson’s disease, *Periplaneta americana*, ROS, ER stress, AKT, Neuroprotective effects

## Abstract

**Background:**

Parkinson’s disease (PD) is a chronic neurodegenerative disorder that currently has no curable strategies. More and more evidence suggests that endoplasmic reticulum (ER) stress plays an essential role in PD pathogenesis. *Periplaneta americana L.* (*P. americana*) is a traditional Chinese medicine with diverse therapeutic properties. This study aims to investigate the neuroprotective effect and underlying mechanism of *P. americana* in in vitro and in vivo PD models.

**Methods:**

The exposure of SH-SY5Y cells to 1-methyl-4-phenyl-pyridinium (MPP^+^) was used as the in vitro PD model. MTT assay, Hoechst staining, Calcein AM-PI staining and flow cytometry were performed to measure the cell viability and apoptosis. DCFH-DA and JC-1 assay were used to measure the intracellular ROS and mitochondrial membrane potential (Δψm), respectively. Western-blot and immunostaining were conducted to detect the expression of key molecules related with ER stress. For the in vivo PD model induced by 1-methyl-4-phenyl-1, 2, 3, 6-tetrahydro-pyridine (MPTP), the motor function of mice was assessed by behavioral tests, the level of TH was examined by western-blot and immunostaining, the expression of key molecules related with ER stress was measured by western-blot.

**Results:**

*Periplaneta americana* ethanol extract (PAE) concentration-dependently inhibited MPP^+^-induced cell loss and increased cell viability. PAE also remarkably attenuated ROS accumulation, the decline of Δψm as well as the excessive ER stress. The neuroprotective effects of PAE could be blocked by ROS inducer trimethylamine N-Oxide or ER stress activator tunicaymycin, while the antioxidant N-Acetyl-L-cysteine or ER stress inhibitor sodium 4-phenylbutyrate mimicked the effects of PAE. Furthermore, we found that PAE could activate AKT/GSK3β/β-catenin pathway. The effect of PAE on ROS production, Δψm and ER stress was blocked by AKT inhibitor MK-2206. In in vivo model, PAE significantly improved motor function, prevented dopaminergic neuronal loss and attenuated ER stress in substantia nigra and striatum of MPTP-treated mice. Similarly, the effects of PAE on MPTP-treated mice were also abolished by MK-2206.

**Conclusions:**

Our results suggest that *P. americana* exerts neuroprotective effects through inhibiting ER stress via AKT-dependent pathway. *Periplaneta americana* may represent a promising therapeutic agent for PD treatment and is worthy of further being exploited.

## Background

Parkinson's disease (PD) is the second most common neurodegenerative disorder globally, with the prevalence increasing sharply over the past three decades [1]. The clinical characteristics of PD mainly consist of motor symptoms and non-motor symptoms. The typical motor features include bradykinesia, muscle rigidity, tremor, and altered gait and postural reflexes. The non-motor symptoms often manifested as olfactory dysfunction, sleep dysregulation (e.g., rapid eye movement sleep behavior disorder), autonomic dysfunction (e.g., dizziness, constipation, and reduced bladder control), neuropsychiatric abnormalities (e.g., anxiety, depression and psychosis) and cognitive decline (particularly impairment of executive function) [2]. Currently, the primary therapies for PD (e.g., dopamine therapy and deep brain stimulation) only relieve the symptoms but cannot slow the progression of PD [3]. On the other hand, side effects remain a challenging issue in the treatment of PD. For example, the dyskinesias, on–off phenomenon, hallucinations and delusions induced by levodopa even worsen the health of PD patients [4]. The PD neuropathology is characterized by misfolded accumulation of α-synuclein and Lewy body formation in the substantia nigra pars compacta (SNpc), which leads to progressive degeneration of dopaminergic neurons in the SNpc [5]. The loss of SNpc dopaminergic neurons finally compromises signaling to the striatum, resulting in nigrostriatal insufficiency and dopaminergic deficiency. The etiology of PD is poorly understood, it appears to be caused by complex interactions of environmental and genetic factors [4]. Multiple mechanisms including genetic mutations [6], incorrect protein folding [7], oxidative stress [8], neuroinflammation [9], mitochondrial dysfunction and gut dysbiosis [8], have been considered to be involved in the loss of dopaminergic neurons during the development of PD. The complex pathogenesis of PD brings great challenges to drug development and reminds us that further exploration of the exact mechanisms of dopaminergic neuron degeneration remain urgent. At the same time, the limitations of current therapies also highlight necessity of developing novel treatments for this disease.

The endoplasmic reticulum (ER) is a large organelle that spreads throughout the cytoplasm and is also the main site for protein biosynthesis, post-translational modification, folding, assembling and maturation [10]. As a membranous compartment, it is highly sensitive to stressors that affect its structure, integrity or function. ER disruption will result in the aggregation of unfolded or misfolded proteins through activating the unfolded protein response, which is an adaptive response (UPR) [10]. However, the prolonged or excessive stress leads to programmed cell death. Due to the hypersensitivity of neuronal cells to protein misfolding, ER stress and UPR dysfunction was reported to be critically involved in neurodegenerative disorders such as Alzheimer’s disease and Parkinson’s disease [10]. UPR is controlled by three ER transmembrane proteins: inositol-requiring 1α (IRE1α), double-stranded RNA dependent protein kinase-like ER kinase (PERK), and activating transcription factor 6 (ATF6) [11]. The 78-kDa glucose-regulated protein (GRP78), also known as binding immunoglobulin protein (BiP), acts as the regulator of UPR initiation through interacting with the three ER sensors mentioned above. Under physiological condition, the binding of these ER transmembrane sensors to GRP78 stabilizes their inactive states. However, under ER stress, GRP78 is released from the luminal domain of above ER proteins, then inducing their functional conformation and activation [12]. UPR triggered by ER sensors activation can attenuate further protein synthesis, which is beneficial for cell survival [13]. However, excessive or prolonged ER stress leads to the overproduction of unfolded or misfolded proteins and then activates programmed cell death. For example, the activated PERK phosphorylates eukaryotic initiation factor 2α (eIF2α) to induce the expression of C/EBP homologous protein (CHOP), consequently triggering cell apoptosis by down-regulating anti-apoptotic molecules and up-regulating pro-apoptotic molecules [14]. The activated IRE1α promotes mitochondrial-mediated apoptosis by stimulating the apoptotic-signaling kinase-1, followed by activation of Jun-N-terminal kinase (JNK) and subsequent cleave of caspase-3 [15]. Hence, the neuroprotective agents that could alleviate the ER stress may exert neuroprotective effects against PD.

The *Periplaneta americana* L. (*P. americana*) is one of the largest insects in the genus *Periplaneta* [16]*.* In the ancient Chinese medicinal book “Shen Nong Ben Cao Jing”, the dry body of P. americana has been recorded as a traditional medicine, usually used to treat ulcer, burn, infantile sore throat, sore carbuncle, insect and snake bite, etc. [17, 18]. Modern pharmacological studies found that *P. americana* extract (PAE) possesses multifunction, including promoting tissue repair and regeneration, exerting antibacterial, antiviral, anti-inflammatory, anticancer and neuroprotective activities [19–22]. For example, it has been shown that PAE attenuates the neurological dysfunction of stroke rats by promoting neurogenesis and angiogenesis via CREB/BDNF signaling pathway [22]. Although there are already some preparations from *P. americana* on the market (e.g. Kangfuxin) to be used for treating ulcerative and inflammatory diseases [23, 24], *P. americana* have not yet been fully investigated, especially its role in nervous system.

In the present study, we aimed to explore whether *P. americana* exerted protective effects against PD models, as well as the role of ER stress in above process. In in vitro experiments, SH-SY5Y cells were exposed to N-Methyl-4-Phenylpyridinium Iodide (MPP^+^) to develop a cellular model of PD [25, 26] and the neuroprotective effects of *P. americana* ethanol extract (PAE) as well as the potential mechanisms were investigated. We also evaluated the influence of PAE on neurological function, dopaminergic neurons in SNpc and striatum, and pathways related with ER stress in mice subjected to 1-Methyl-4-phenyl-1, 2, 3, 6- tetrahydropyridine (MPTP) in vivo [25]. The results of our study suggest that *P. americana* has promising therapeutic potential in the treatment of PD.

## Materials and methods

### Materials

N-Methyl-4-Phenylpyridinium Iodide (MPP^+^), Trimethylamine N-Oxide (TMAO), Tunicaymycin, Sodium 4-Phenylbutyrate (4-PBA) were obtained from Aladdin Inc (Shanghai, China), 1-Methyl-4-phenyl-1, 2, 3, 6- tetrahydropyridine (MPTP) and MK-2206 2HCL were purchased from MACKLIN (Shanghai, China). Dulbecco's modified Eagle's medium (DMEM)/Nutrient Mixture F-12 (DMEM/F12), 0.25% trypsin, penicillin–streptomycin and fetal bovine serum (FBS) were purchased from Gibco (Grand Island, NY). The primary antibody against CHOP, AKT, p-AKT (Ser473), p-GSK-3β (Ser9) was purchased from Cell Signaling Technology (MA, USA). Anti-GSK-3β antibody was from Abcam (Cambridge, MA, USA). The primary antibody against β-catenin, JNK, p-JNK, Caspase-3/Cleaved caspase-3, Bax, Bcl-2, Tyrosine Hydroxylase (TH) were from WanleiBio (Shenyang, China). Hoechst 33,342, 3-(4,5-dimethyl-2-thiazolyl)-2,5-diphenyl-2-H-tetrazoliumbromide (MTT), the kits of5,5′,6,6-tetrachloro-1,1′,3,3′-tetraethylbenzimidazolyl-carbocyanineiodide (JC-1), Pi-dium (PI), and DCFH-DA, N-Acetyl-L-cysteine (NAC) and the primary antibody against IRE-1α, p-IRE-1α, eIF-2α, p-eIF-2α, GRP78 were from Beyotime Biotechnology (Shanghai, China). The primary antibodies against β-actin and all of the secondary antibodies were obtained from ProteinTech Inc (Wuhan, China). The reagents used in this research were all commercially available and meet biochemical quality standards.

### *Periplaneta americana* L. extract preparation

The dry body of *Periplaneta americana L.* (*P. americana*) was from GoodDoctor Pharmaceutical Group (Sichuan province, China), identified by characteristics and confirmed to be genuine by thin-layer chromatography (TLC). Specifically, it is reddish brown in color and has wings longer than the end of its abdomen and a large butterfly shaped brown stripe in the middle of the pronotum from appearance. The sample and the reference substance display the same spots at the corresponding positions when identified by TLC. To prepare the extract for experimental use, *P. americana* was ground to powder and degreased in petroleum ether for 24 h. After removing the petroleum ether completely, added 75% ethanol to the degreased powder until it swelled, and then transferred it to a percolation tube to percolate extraction within five-fold times of 75% ethanol for 48 h. The flow rate was controlled at 3 mL/min. The ethanol extract was collected and centrifuged at 12,000 rmp for 10 min, and the supernatant was filtrated through 0.45 μm microporous membrane. The filtered extract was then evaporated under reduced pressure followed by lyophilization to produce dried extract with yield of 15.7%.

### LC–MS/MS conditions

The Agilent ultra-high performance liquid chromatography 1290 UPLC system with ACQUITY UPLC® HSS T3 (2.1 × 100 mm, 1.8 µm) chromatographic column (Waters, Milford, MA, USA) was used to perform the LC–MS/MS analyses. The flow rate was controlled at 0.3 mL/min, the temperature was set at 40 ◦C and the injection volume was 2 μL. The mobile phase consisted of 0.1% formic acid in acetonitrile (B1) and 0.1% formic acid in water (A1) under positive ions mode. The multi-step linear elution gradient program was: 0–1 min, 8% B1; 1–8 min, 8%-98% B1; 8–10 min, 98% B1; 10–10.1 min, 98%–8% B1; 10.1–12 min, 8% B1. Under negative ions mode, the mobile phase is acetonitrile (B2) and 5 mM ammonium formate in water (A2). The multi-step linear elution gradient program was: 0–1 min, 8% B2; 1–8 min, 8%-98% B2; 8–10 min, 98% B2; 10–10.1 min, 98% -8% B2; 10.1–12 min, 8% B2.

A Thermal Q Exactive Focus mass spectrometer together with the Xcalibur software (version 4.1) was used to get MS and MS/MS data. During each acquisition cycle, the mass range was from 100 to 1500, and the top three for each cycle were screened, with corresponding MS/MS data acquisition. Spray Voltage: 3.5 kV (positive) or -2.5 kV (negative). Sheath gas flow rate: 40 arb, Aux gas flow rate: 10 arb, Capillary temperature: 325 °C, Full scan resolution: 70,000. HCD was used for secondary fragmentation with a collision energy of 30 eV, MS/MS resolution: 17,500.

### Cell culture

The SH-SY5Y cell line used in our study was from American Type Culture Collection (ATCC, USA). The cells were cultured in flasks containing DMEM/F12 modified with 10% FBS and 1% penicillin/streptomycin, and maintained in an incubator at 37 °C in 95% air/5% carbon dioxide. The cells were passaged at 48 h per interval and seeded into 96-well plates, 24-well plates or 6-well plates according to the experiment design.

### Cell viability analysis

Cell viability was determined by the MTT assay. The SH-SY5Y cells were cultured in 96-well plates at a density of 5 × 10^3^ cells/well for 24 h. Then, the culture medium was discarded and new medium containing different concentrations of MPP^+^ or PAE was added for another 48 h. To study the effect of PAE on MPP^+^ toxicity, the cells were pretreated with various concentration of PAE for 2 h, followed by exposure to MPP^+^ for another 48 h. In the latter experiments, for the cells needed to be treated with TMAO or tunicaymycin, they were added to the wells simultaneously with PAE. For the cells needed to be treated with NAC or 4-PBA, added these compounds into the well for 2 h and then exposed the cells to MPP^+^ (500 μM) for another 48 h. Subsequently, the DMEM medium containing MTT (0.5 mg/mL) was added to each well and then incubated at 37 °C for 4 h. The medium was then replaced by 200 μL dimethyl sulfoxide (DMSO) to dissolve the formazan product. The optical density (OD) was measured at 570 nm with a multi-functional microplate reader (Synergy HT, Bio-TEK instruments Inc., USA). The cell viability was expressed as a percentage of the absorbance of untreated cultures.

### Hoechst 33,258 staining

The cells were cultured in 48-well plates at a density of 2 × 10^4^ cells/well. They were pretreated with different concentrations of PAE for 2 h and then incubated for another 48 h in the presence or absence of MPP^+^ (500 μM). For the cells needed to be treated with TMAO, tunicaymycin, NAC or 4-PBA, added them to the well in the way as described above. The cells were then fixed with 4% paraformaldehyde for 20 min at room temperature. After being washed by PBS, the cells were stained with Hoechst 33,258 (10 μg/mL) for 15 min, and the nuclear changes were observed with an inverted fluorescence microscope (Nikon, Japan) [27].

### Calcein AM-PI staining

The Calcein AM-PI Staining Kit (Beyotime Biotech, Shanghai, China) was used to detect live and dead cells [27]. Briefly, SH-SY5Y cells were pretreated with PAE for 2 h and incubated with or without MPP^+^ (500 μM) for another 48 h. For the cells needed to be treated with TMAO, tunicaymycin, NAC or 4-PBA, added them to the well in the way as described above. After the treatment, cells were then stained with Calcein AM-PI solution for 20 min at room temperature. After being washed with PBS, the fluorescence was detected with an inverted fluorescence microscope.

### Flow cytometric analysis

Flow cytometric analysis with Annexin V-FITC/PI double staining was performed to assess the cell apoptosis [28]. Briefly, SH-SY5Y cells were seeded in 6-well plates and treated as described above. Subsequently, the cells were digested and centrifuged at 1000 rpm for 5 min. Afterwards, the cells were suspended with 500 μL binding buffer and incubated in dark with Annexin V-FITC and PI for 15 min at room temperature. Cell fluorescence was analyzed by a flow cytometer (BD FACSVerse™ flow cytometer, BD Biosciences, San Jose, CA, USA). Viable cells, early apoptotic cells, late apoptotic cells and cellular debris were identified as Annexin V^−^/PI^−^, Annexin V^+^/PI^−^, Annexin V^+^/PI^+^ and Annexin V^−^/PI^+^, respectively.

### Intracellular ROS measurement

Cells were cultured in 24-well plates at the density of 2 × 10^4^ cells per well. The treatment schedule was same as described above. For the cells needed to be treated with MK-2206, 5 μM MK-2206 2HCL was added to each well simultaneously with PAE. After the treatment, the cells were incubated with DCFH-DA (10 μM) for 20 min at 37 °C in dark [29]. Subsequently, cells were washed with PBS and the fluorescence of DCFH-DA was imaged under a fluorescence microscope (Nikon, Tokyo, Japan).

### Mitochondrial membrane potential measurement

The mitochondrial membrane potential (Δψm) was measured by JC-1 kit (Beyotime Biotech) [30]. JC-1 is a cell-permeant dye that can accumulate within mitochondria. It is widely used to detect Δψm. In normal cells, JC-1 aggregates in the mitochondrial matrix to form J-aggregates and produce red fluorescence. However, in the apoptotic cells, Δψm is decreased and JC-1 cannot be gathered in the mitochondrial matrix, thus JC-1 exists in the form of a monomer and produces green fluorescence [31]. The ratio of red/green fluorescence can be used to reflect the change of Δψm. As described above, the cells were pretreated with various concentration of PAE for 2 h and then exposed to MPP^+^ for another 48 h. For the cells needed to be treated with MK-2206, it was added to each well simultaneously with PAE at a final concentration of 5 μM. Afterwards, the cells were washed and incubated with JC-1 staining solution for 20 min at 37 °C in dark. Finally, the cells were washed with JC-1 staining buffer three times and the fluorescence was imaged with an inverted fluorescence microscope (Nikon, Tokyo, Japan). For red fluorescence, the excitation and emission light was 525 nm and 590 nm. For green fluorescence, the excitation and emission light was 490 nm and 530 nm, respectively.

### Immunofluorescence staining

Cells were fixed with 4% paraformaldehyde for half an hour followed by PBS washing, and then blocked with 10% goat serum containing 0.3% Triton X-100 for half an hour at room temperature [29]. Afterwards, the cells were incubated with the rabbit anti-p-IRE1α antibody (1:200), anti-p-eIF2α antibody (1:200), anti-GRP78 antibody (1:200) and anti-CHOP antibody (1:200) at 4 °C overnight. On the next day, the cells were washed by PBS for three times and then incubated with Cy3-labeled goat anti-rabbit IgG (1:100) at room temperature in the dark for 1 h. After removing the secondary antibody and washing with PBS, the cells were stained with DAPI for 15 min. Cell fluorescence was imaged and captured using a confocal microscope. Six views of each group were selected randomly and used for the following analysis.

For the immunostaining of brain tissues in the latter experiment, the procedure was similar to that of cultured cells. In brief, the mouse brain was removed rapidly after perfusion and post-fixed overnight in 4% paraformaldehyde at 4 °C. Subsequently, the tissue was dehydrated sequentially with 20% and 30% sucrose solution until it sank to the container bottom. The coronal sections (15 μm) containing striatum and substantia nigra were obtained using a freezing microtome (Leica). After being washed with PBS for three times, the cryosections were blocked with 10% goat serum containing 0.3% Triton X-100 at room temperature for 1 h. Then sections were incubated with primary antibodies overnight in 10% normal goat serum at 4 °C. Subsequently, the sections were rinsed thress times with PBS and then incubated with the fluorescence-labeled secondary antibody in dark for 1 h at room temperature. The sections were washed again and stained with DAPI for 15 min. Finally, the sections were coverslipped with 50% glycerine and photographed under a microscope.

### Animal experiments

Male C57BL/6 mice (8 weeks old, specific pathogen free) were obtanied from Slike Jingda Laboratory Animal Co., Ltd (Hunan, China). The mice were housed under standard laboratory conditions (temperature 22–26 °C, humidity 50–70%, 12 h light/dark cycle) with water and food provided ad libitum. Before the experiments, they were allowed to acclimate to laboratory environment for one week. All experiments were conducted in accordance with the guidance for the Care and Use of Laboratory Animals published by the US National Institutes of Health [32] and the protocols approved by Animal Care and Use Committee of the Southwest University.

Mice were randomly divided into four groups: the vehicle group, the MPTP group, the MPTP + PAE group, the MPTP + PAE + MK2206 group. Mice in the vehicle group and MPTP group were administered with saline intragastrically (i.g.), and the other two groups were given the same volume of PAE (120 mg/kg, dissolved in saline, i.g.) from day 1 to day 14. From the third day to the seventh day, two hours after the administration of saline or PAE, the mice in the vehicle group were given saline intraperitoneally (i.p.) (10 mL/kg) and mice in the other three groups were given the same volume of MPTP (30 mg/kg, i.p.). For mice in the MPTP + PAE + MK2206 group, the Akt inhibitor MK-2206 2HCl (100 mg/kg, i.g.) was administrated every other day starting from day 3, for a total of six times. Behavioral tests were carried out on day 15, and then the mice were sacrificed by overdosing 5% isoflurane and the tissues were collected for the following experiments.

### Rotarod test

The rotarod test is commonly used to assess the motor coordination and balance in rodents [29]. Mice were placed on a rotarod rod (Ji-Nan Yiyan company) and the rolling speed was adjusted so that it gradually increased to 30 rpm within 30 s. The latency of each mouse to fall off the rotarod was recorded. The maximum cutoff time was 180 s. If the animal had not fallen after 180 s, the latency would also be recorded as 180 s. Each mouse was tested for three times with an interval time of at least half an hour. To reach optimal performances, the mice were subjected to three consecutive days of adaptive training before the formal experiment.

### Pole test

The pole test was commonly used to detect the coordination ability of mice [33]. A straight wooden rod with the diameter of 0.8 cm and height of 50 cm was used in the pole test. A small wooden ball wrapped with bandage gauze was placed at the top of the rod to prevent the mouse from slipping [29]. Mice were trained in three test trials for two days prior to the formal experiment. In the formal test, mouse was placed on top of the straight rod, and the time it took to completely turn around and face the floor was recorded as T-turn (time to turn) and the total time required to arrive at the bottom of rod was recorded as T-down (time to climb down). Each mouse was tested three times with an interval of at least half an hour.

### Western blot analysis

Western blot analysis was carried out as previously described [34]. In brief, the harvested cells or brain tissues (striatum and substantia nigra) were lysed with RIPA buffer containing the proteinase and phosphatase inhibitors. The samples were subjected to an ultrasonic cell disruptor in order to be homogenized completely, and then centrifuged at 12,000 rpm for 15 min at 4 °C. The supernatant was collected and the protein concentration was quantified with BCA Protein Assay Kit (Beyotime Biotech). Equal amounts of protein (30 μg) were separated by SDS-PAGE gel and then electrotransferred onto PVDF membranes (Invitrogen). The PVDF membranes were blocked in 5% fat-free milk at room temperature for 2 h, then the bands were cut according to their molecular weight and incubated overnight with primary antibodies at 4 °C. Subsequently, the membranes were washed three times with TBST for 10 min each time, and then probed with the horseradish peroxidase-conjugated anti-mouse or anti-rabbit IgG secondary antibodies for 1.5 h at room temperature. Finally, the bands were visualized using an enhanced chemiluminescence (ECL) system (Perkin Elmer). The results were expressed the ratio relative to the band intensity of control group.

### Statistical analysis

Results were presented as means ± *SD*. Statistical comparisons were performed with GraphPad Prism 7 (San Diego, CA, USA) using one-way analysis of variance (ANOVA) followed by Tukey’s test for post hoc comparisons. In all cases, *p* < 0.05 was considered as statistically significant.

## Results

### PAE protected SH-SY5Y cells against the cytotoxicity induced by MPP^+^

**Fig. 1:**
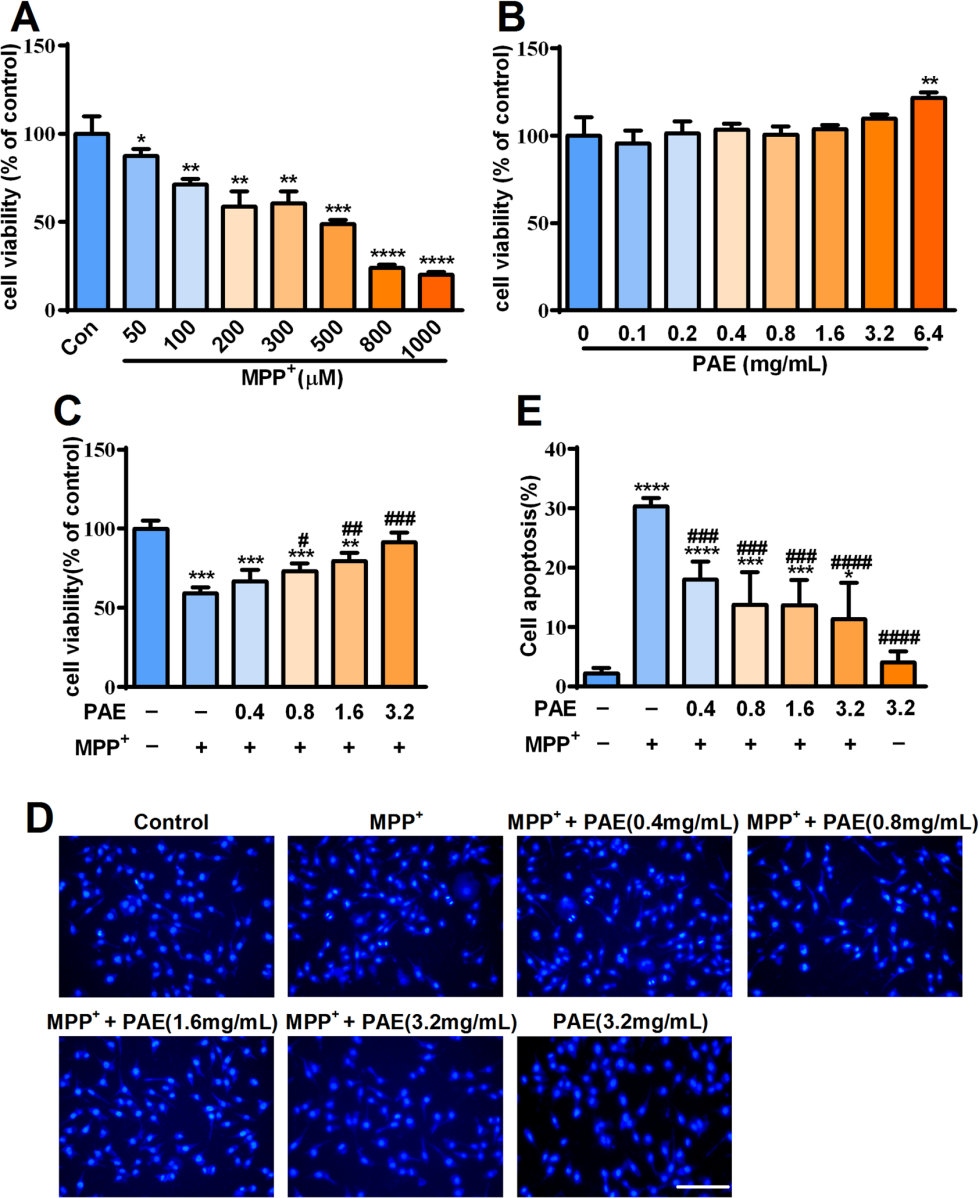
Effects of MPP^+^ and ethanol extract of *Periplaneta americana L.* (PAE) on SH-SY5Y cell viability. **A** The cell viability of SH-SY5Y incubated with different concentrations of MPP^+^ ranging from 50 μM to 1000 μM for 48 h. **B** The cell viability of SH-SY5Y treated with different concentrations of PAE for 48 h. **C** Cells were pretreated with different concentrations of PAE for 2 h followed by incubation with 500 μM MPP^+^ for another 48 h. Cell viability was expressed as the percentage of the viability of the control group. **D** Cells were pretreated with different concentrations of PAE for 2 h followed by incubation with or without MPP^+^ (500 μM) for another 48 h, apoptotic cells were evaluated by hoechst staining, bar = 100 μm. **E** Quantification of apoptotic cells. Data are presented as mean ± SD (n = 3), ^*^p < 0.05, ^**^p < 0.01, ^***^p < 0.001, ^****^p < 0.0001, *vs.* control group, ^#^*p* < 0.05, ^##^*p* < 0.01, ^###^*p* < 0.001, ^####^*p* < 0.0001, *vs.* MPP^+^ treated group

**Fig. 2:**
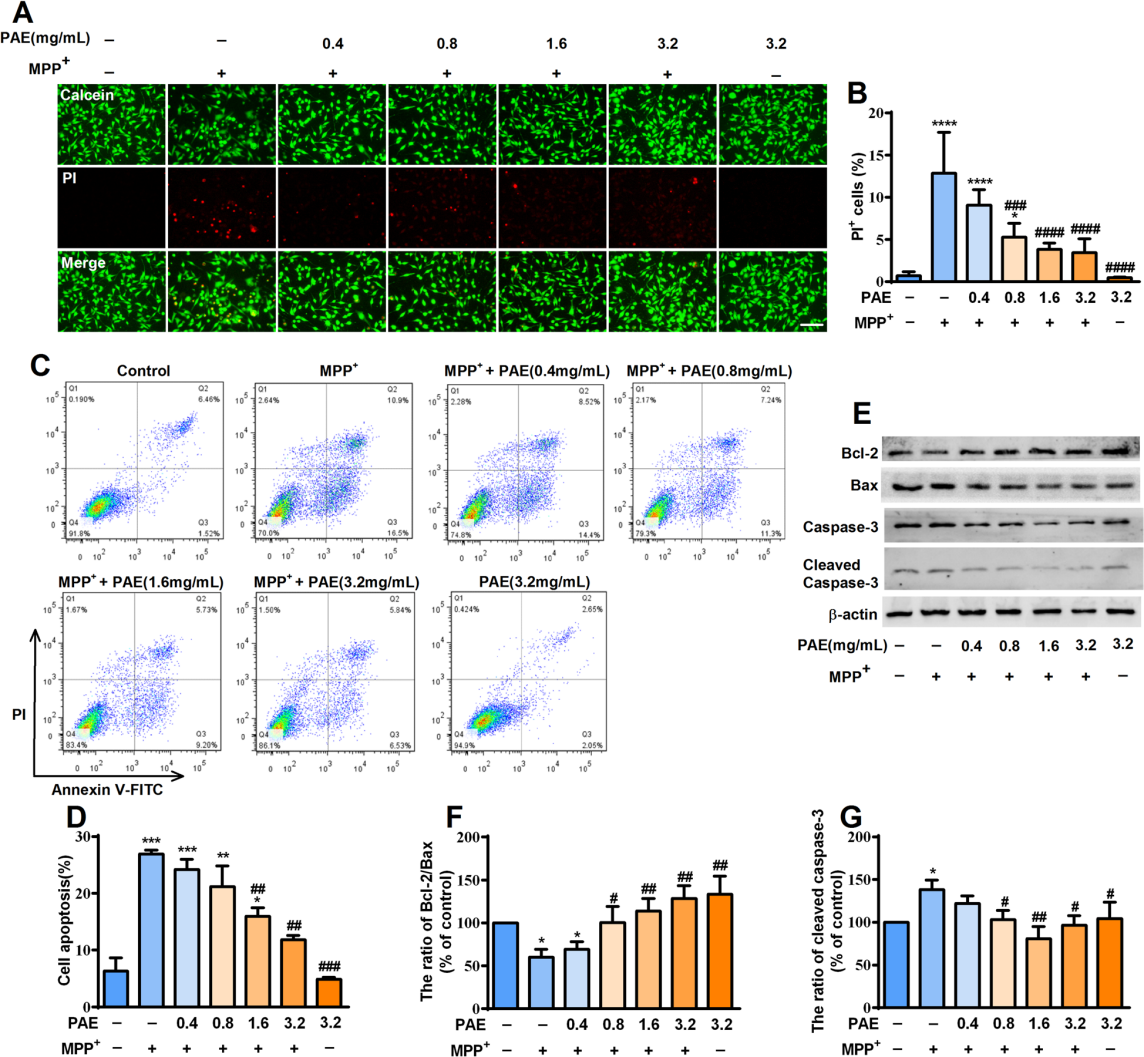
PAE prevents cell loss induced by MPP^+^ in SH-SY5Y cells. **A**, **B** Cells were pre-incubated with PAE for 2 h followed by treating with or without MPP^+^ (500 μM) for another 48 h, Calcein-PI staining was performed to evaluate the role of PAE on cell death (bar = 100 μm). **C**, **D** Cell apoptosis was evaluated by flow cytometry. **E**–**G** Expression of Bcl-2, Bax and cleaved caspase-3 was assessed by Western blot. PAE pretreatment attenuated the reduction of Bcl-2/Bax ratio and up-regulation of cleaved caspase-3 induced by MPP^+^ (n = 3). Data are presented as mean ± SD, ^*^p < 0.05, ^**^p < 0.01, ^***^p < 0.001, ^****^p < 0.0001, *vs.* control group, ^#^*p* < 0.05, ^##^*p* < 0.01, ^###^*p* < 0.001, ^####^*p* < 0.0001, *vs.* MPP^+^ treated group

Exposing SH-SY5Y cells to MPP^+^ is widely used as in vitro model for studying PD. We firstly assessed the cytotoxicity of MPP^+^ and found that it reduced the cell viability of SH-SY5Y cells in a concentration-dependent manner after incubating for 48 h. The cell viability was reduced by nearly half at 500 μM measured by MTT assay (Fig. 1A). Thus, this concentration (500 μM) of MPP^+^ was used in the following experiments. We also examined the effect of PAE alone on SH-SY5Y cells and found that treatment with PAE (0.1–3.2 mg/mL) alone had no obvious effects on cell viability (Fig. 1B). To evaluate whether PAE has a protective effect against MPP^+^-induced cytotoxicity, the SH-SY5Y cells were pretreated with PAE (0.4–3.2 mg/mL) for 2 h and then exposed to MPP^+^ (500 μM) for 48 h. As shown in Fig. 1C, PAE attenuated the cell loss induced by MPP^+^ in a concentration-dependent manner. Hoechst 33,258 staining was used to evaluate the morphological change, and nuclear condensation was observed in the MPP^+^-treated cells indicated by a bright fluorescence (Fig. 1D). Pretreatment with PAE concentration-dependently reduced the numbers of apoptotic cells caused by MPP^+^ (Fig. 1E). To further confirm the protective effects of PAE, calcein/PI double staining and flow cytometry were used to detect cell apoptosis. The result of calcein/PI staining suggested that MPP^+^ caused a significant increase in PI-positive cells, whereas pretreatment with PAE markedly reduced the ratio of PI-positive cells (Fig. 2A, B). In flow cytometry analysis, viable cells, early apoptotic cells, late apoptotic cells and cellular debris were identified as Annexin V^−^/PI^−^, Annexin V^+^/PI^−^, Annexin V^+^/PI^+^ and Annexin V^−^/PI^+^, respectively. As shown in Fig. 2C, D, MPP^+^ treatment resulted in an evidently increase in number of apoptotic cells, while PAE pre-incubation inhibited the effect of MPP^+^. To further verify the function of PAE, western blot was performed to examine the expression of apoptosis-related proteins. Bax is a pro-apoptotic molecule which can translocate to the mitochondria and forms dimers, then leading to the release of cytochrome c and activation of caspase family proteins (such as caspase-3). Cleaved caspase-3 is the executor of apoptosis, while overexpression of anti-apoptotic Bcl-2 is supposed to counter these effects [35]. As shown in Fig. 2E–G, MPP^+^ insult led to a increase in the level of bax and cleaved caspase-3 while resulted in a reduction in the level of Bcl-2. PAE pretreatment inhibited MPP^+^-induced decrease in Bcl-2/Bax ratio and increase in cleaved caspase-3 (Fig. 2E–G). Taken together, these results suggest that PAE is neuroprotective against the cytotoxicity of MPP^+^ in SH-SY5Y cells.

### PAE inhibited the accumulation of intracellular ROS and reduction of Δψm triggered by MPP^+^ in SH-SY5Y cells

**Fig. 3:**
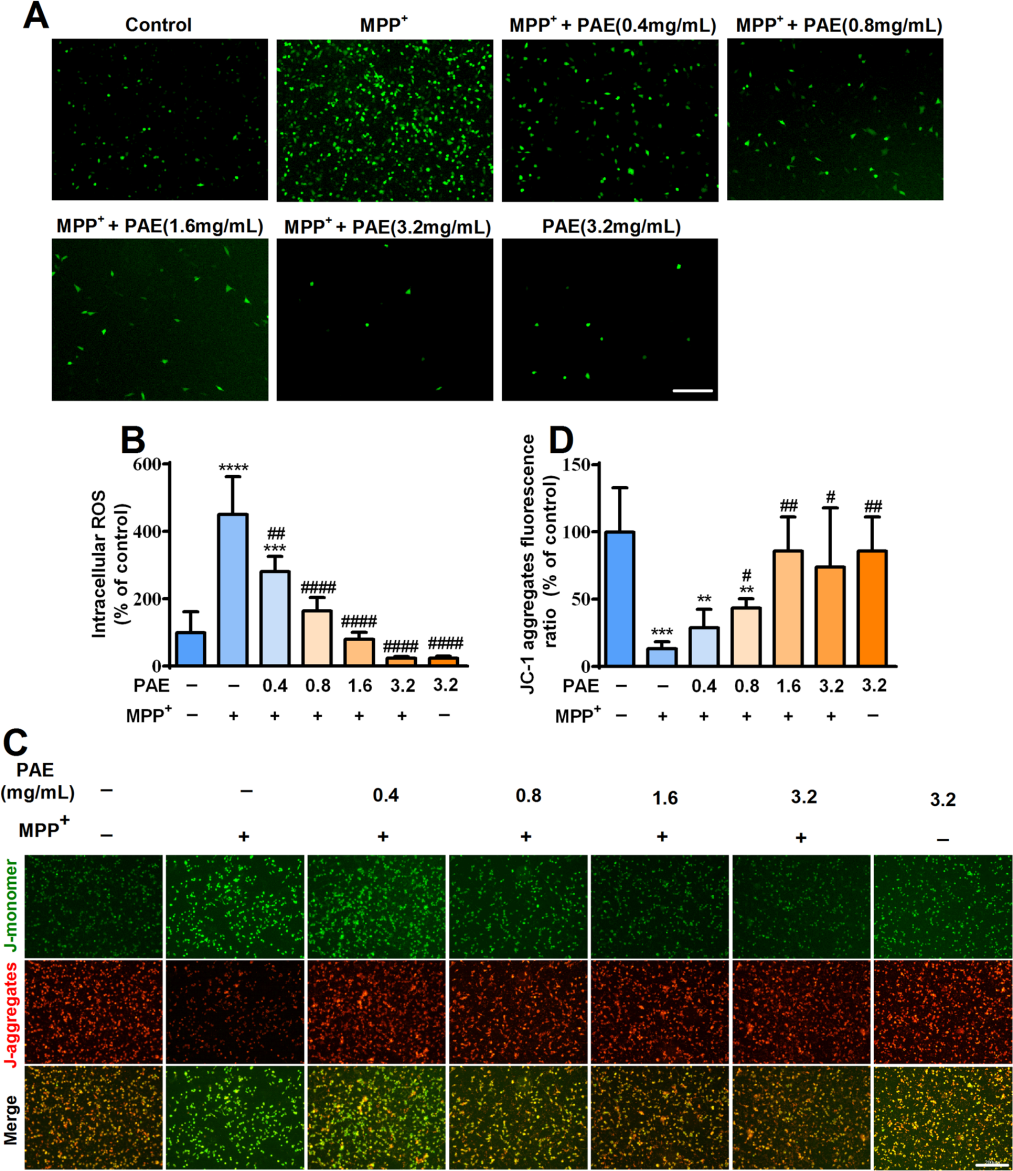
PAE inhibits intracellular ROS accumulation and mitochondrial membrane potential (Δψm) loss induced by MPP^+^. **A** After pre-incubation with different concentrations of PAE for 2 h, SH-SY5Y cells were treated with or without MPP^+^ (500 μM) for another 48 h, the level of ROS in each group were assessed by DCFH-DA assay, bar = 200 μm. **B** PAE prevented the accumulation of intracellular ROS caused by MPP^+^ in a concentration-dependent manner (n = 3). **C** JC-1 staining was performed to detect the Δψm in each group, bar = 200 μm. **D** The statistical results of JC-1 aggregates fluorescence ratio in each group (n = 3), PAE attenuated the reduction of JC-1 aggregates ratio induced by MPP^+^ in a concentration-dependent manner. Data are expressed as mean ± SD, ^**^p < 0.01, ^***^p < 0.001, ^****^p < 0.0001, *vs.* control group, ^#^*p* < 0.05, ^##^*p* < 0.01, ^####^*p* < 0.0001, *vs.* MPP^+^ treated group

Mitochondrial dysfunction has been proposed to be critically involved in the progressive dopaminergic neurodegeneration [36]. Mitochondria are the major source of ROS, the overproduction of ROS in mitochondria can disrupt normal redox signaling and destroy reduction–oxidation (REDOX) activity, ultimately lead to mitochondrial dysfunction [31, 37]. DCFH-DA fluorescence assay was used to evaluate the level of ROS in SH-SY5Y cells. As shown in Fig. 3A, B, MPP^+^ treatment dramatically increased intracellular ROS production in SH-SY5Y cells, while PAE pretreatment remarkably suppressed MPP^+^-induced ROS accumulation (Fig. 3A, B). These results suggested that PAE could prevent ROS overproduction in SH-SY5Y cells.

The decrease of Δψm is an indicator of mitochondrial dysfunction. MPP^+^ has been shown to disrupt mitochondrial REDOX activity and Δψm of dopaminergic cells [38]. Therefore, we assessed the Δψm in SH-SY5Y cells with JC-1 staining kits. Compared with the vehicle group, MPP^+^ led to a markedly decrease in the ratio of red to green fluorescence, whereas PAE pretreatment inhibited the effects of MPP^+^ and increased the ratio of red/green fluorescence in a concentration-dependent manner (Fig. 3C, D). These results suggest that PAE can restore the reduced mitochondrial membrane potential caused by MPP^+^.

### PAE attenuated the endoplasmic reticulum stress triggered by MPP^+^ in SH-SY5Y cells

**Fig. 4:**
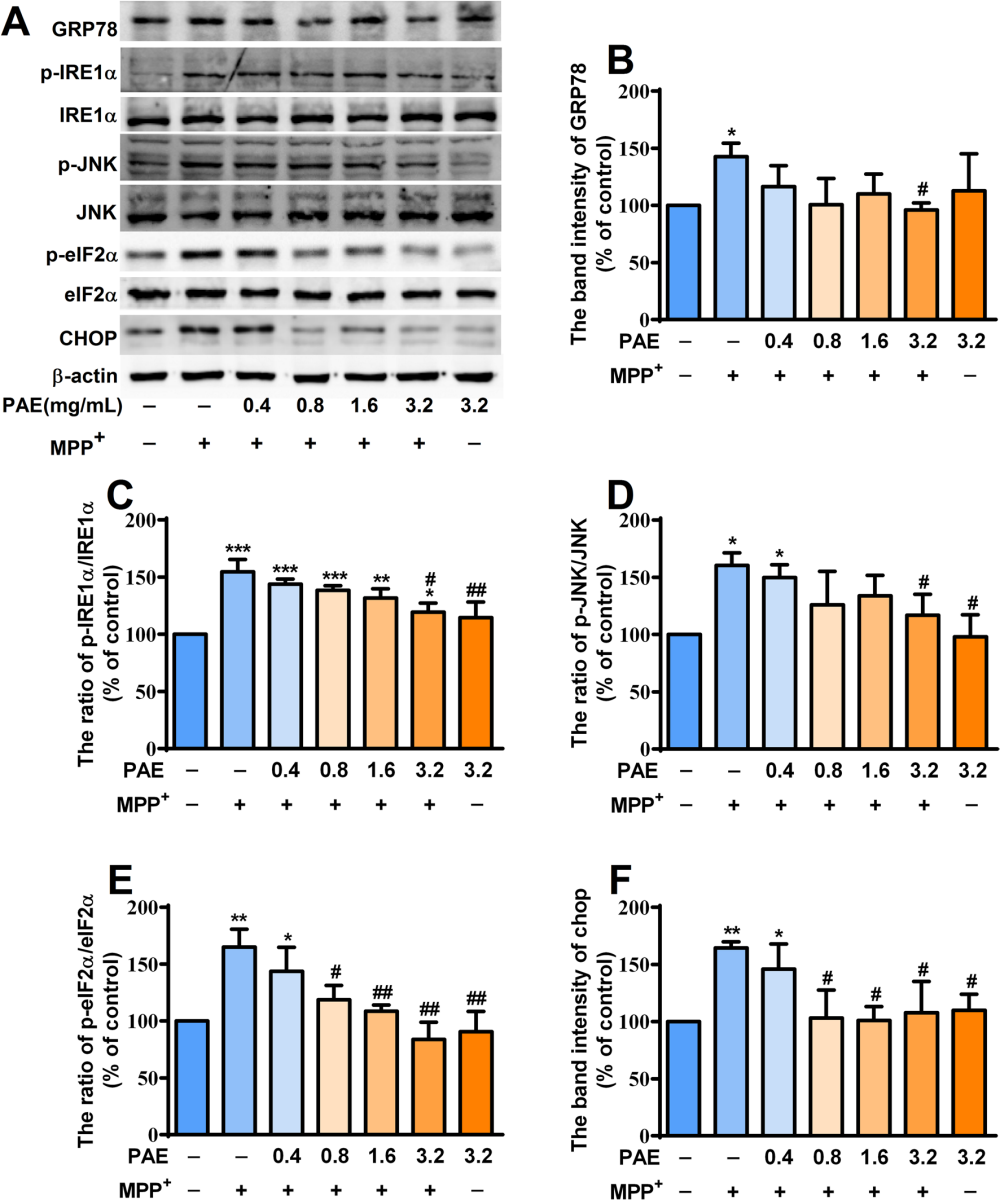
PAE attenuates ER stress in MPP^+^-treated SH-SY5Y cells. **A** After pre-incubation with different concentrations of PAE for 2 h, SH-SY5Y cells were treated with or without MPP^+^ (500 μM) for another 48 h, the protein levels of GRP78, p-IRE1α, IRE1α, p-JNK, JNK, p-eIF2α, eIF2α and CHOP were determined by Western blot. PAE treatment reversed MPP^+^-caused up-regulation of GRP78 (**B**), p-IRE1α/IRE1α ratio (**C**), p-JNK/JNK ratio (**D**), p-eIF2α/eIF2α ratio (**E**) and CHOP (**F**). Results are expressed as mean ± SD, n = 3, ^*^p < 0.05, ^**^p < 0.01, ^***^p < 0.001, *vs.* control group, ^#^*p* < 0.05, ^##^*p* < 0.01, *vs.* MPP^+^ treated group

Endoplasmic reticulum (ER) stress is a normal physiological activity of cells in response to external stimuli, which is beneficial for maintaining cell homeostasis. However, excessive ER stress is an important cause of cell damage and loss, which is closely related to the progression of Parkinson's disease [39]. ROS is a strong inducer of ER stress. Having known that PAE could prevent the accumulation of intracellular ROS, we then evaluated whether it could alleviate the ER stress caused by MPP^+^. The expression of the key molecules involved in ER stress was investigated by western blot. MPP^+^ exposure significantly increased the level of GRP78 (Fig. 4A, B), p-IRE1α (Fig. 4A, C), p-JNK (Fig. 4A, D), p-eIF2α (Fig. 4A, E) and CHOP (Fig. 4A, F) in SH-SY5Y cells. PAE pretreatment suppressed MPP^+^-induced up-regulation of above ER-stress-associated proteins. These results show that PAE can attenuate the ER stress triggered by MPP^+^ in SH-SY5Y cells.

### PAE protected SH-SY5Y cells probably through inhibiting ROS overproduction and ER stress

**Fig. 5:**
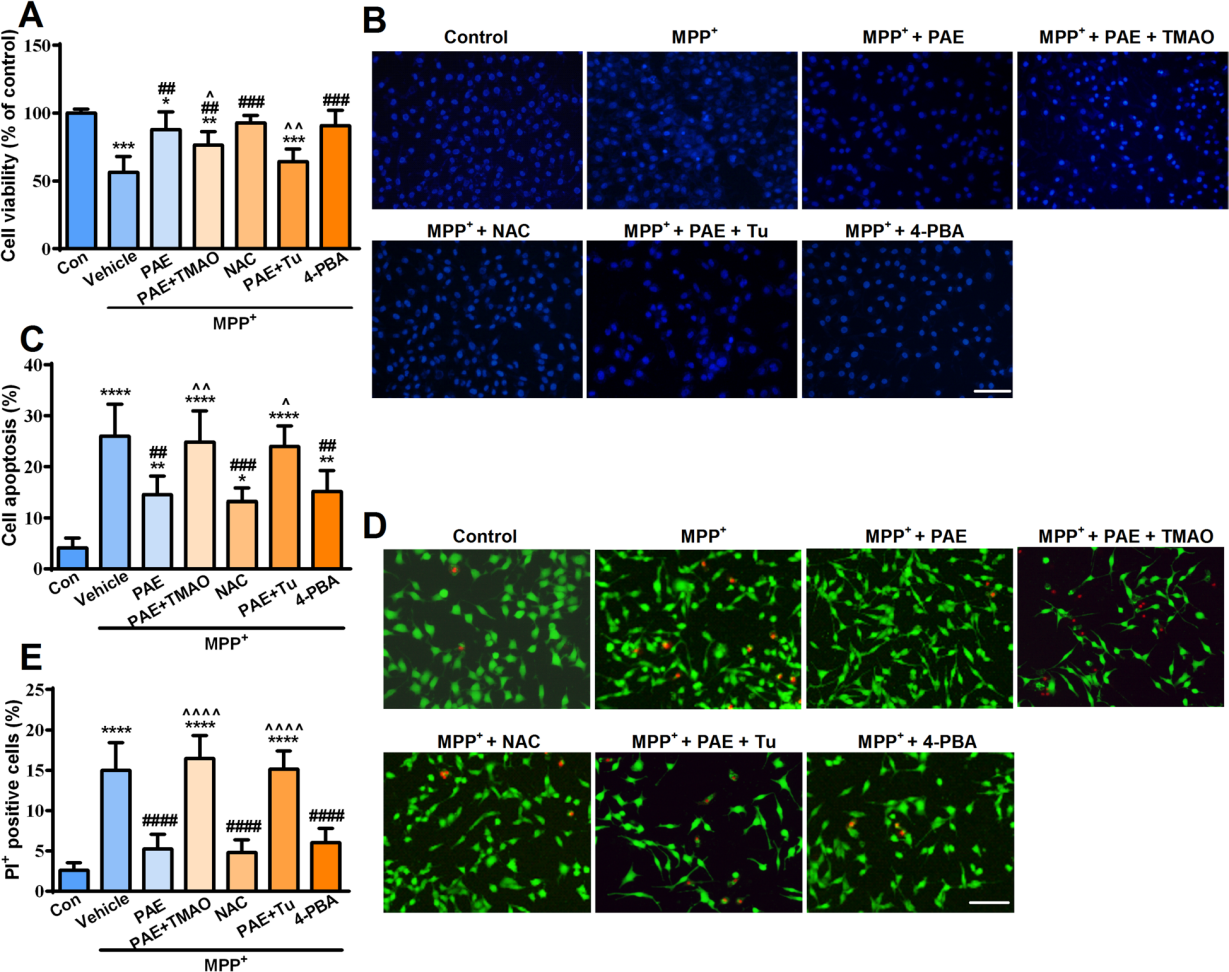
Attenuation of ROS overproduction and ER stress contributes to the neuroprotective effects of PAE. **A** ROS inducer trimethylamine N-Oxide (TMAO) or ER stress activator tunicaymycin (Tu) blocked the effect of PAE (1.6 mg/mL) on cell viability determined by MTT assay, while the antioxidant N-Acetyl-L-cysteine (NAC) or the ER stress inhibitor sodium 4-phenylbutyrate (4-PBA) mimicked the effect of PAE (1.6 mg/mL) on cell viability. Hoechst (B and C) and Calcein-PI staining (D and E) was performed to assess the cell loss in each group (bar = 100 μm). PAE inhibited the cell loss caused by MPP^+^, whereas TMAO or tunicaymycin attenuated the effects of PAE. NAC or 4-PBA mimicked the effects of PAE (B-E). Results are expressed as mean ± SD, n = 3, ^*^p < 0.05, ^**^p < 0.01, ^***^p < 0.001, ^****^p < 0.0001, *vs.* control group, ^##^*p* < 0.01, ^###^*p* < 0.001, ^####^*p* < 0.0001, *vs.* MPP^+^ treated group, ^^^*p* < 0.05, ^^^^*p* < 0.01, ^^^^^^*p* < 0.0001, *vs.* MPP^+^ + PAE group

To explore whether PAE protect SH-SY5Y cells against MPP^+^ through inhibiting ROS overproduction and ER stress, we added TMAO (ROS inducer), NAC (a free radical scavenger), tunicaymycin (ER stress inducer), 4-PBA (ER stress inhibitor) to the wells in the way as described in the “Method” section. We found that the protective effects of PAE (1.6 mg/mL) could be attenuated by TMAO (50 mM) or tunicaymycin (2 μg/mL) measured by MTT assay. In contrast, NAC (2 mM) or 4-PBA (5 mM) both could increase the cell viability and exert neuroprotective effects in SH-SY5Y cells, which mimicked the activity of PAE (Fig. 5A). To further confirm the role of ROS and ER stress in the protective effects of PAE, Hoechst 33,258 and Calcein AM-PI staining were performed. As shown in Fig. 5B, C, PAE (1.6 mg/mL) suppressed the cell apoptosis caused by MPP^+^ insult, while either TMAO or tunicaymycin could block the effect of PAE. Consistently, the number of PI-positive cells decreased significantly in PAE treatment group (1.6 mg/mL), whereas it increased dramatically in the group co-administrated with TMAO or tunicaymycin (Fig. 5D, E). NAC or 4-PBA showed similar protective effects with PAE detected by Hoechst and Calcein AM-PI staining. These data suggest that PAE protect SH-SY5Y cells against MPP^+^ probably through inhibiting ROS overproduction and ER stress.

### PAE activated AKT/ GSK3β/β-catenin pathway in SH-SY5Y cells

**Fig. 6:**
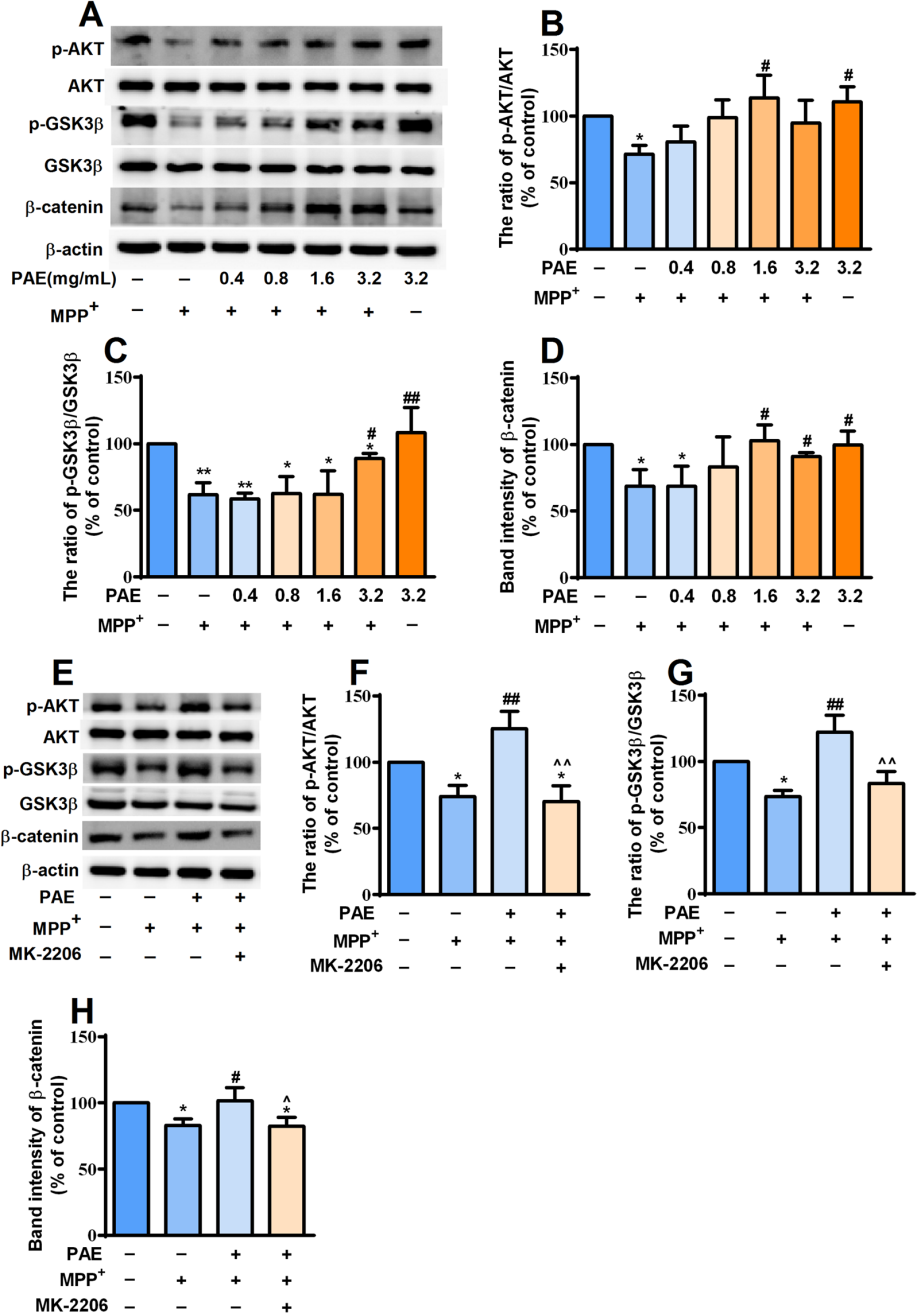
PAE can activate AKT/ GSK3β/β-catenin pathway in SH-SY5Y cells. **A** After pre-incubation with different concentrations of PAE for 2 h, SH-SY5Y cells were treated with or without MPP^+^ (500 μM) for another 48 h. The expression of p-AKT, AKT, p-GSK3β (Ser9), GSK3β and β-catenin was determined by western-blot. PAE treatment reversed MPP^+^-induced down-regulation of p-AKT/AKT ratio (**B**), p-GSK3β/GSK3β ratio (**C**) and β-catenin (**D**). The AKT inhibitor, MK-2206, blocked the effect of PAE on p-AKT/AKT ratio (**E**, **F**), p-GSK3β/GSK3β ratio (**E**, **G**) and β-catenin (**E**, **H**). Results are presented as mean ± SD, n = 3, ^*^p < 0.05, ^**^p < 0.01, *vs.* control group, ^#^*p* < 0.05, ^##^*p* < 0.01, *vs.* MPP^+^ treated group, ^^^*p* < 0.05, ^^^^*p* < 0.01, *vs.* MPP^+^ + PAE group

The activation of AKT related pathways are essential for cell survival. Glycogen synthase kinase-3β (GSK3β)/β-catenin signaling pathway is demonstrated to be regulated by the activation of AKT [40]. We then investigated whether PAE had any effect on AKT/GSK3β/β-catenin pathway. MPP^+^ exposure significantly decreased the level of p-AKT, p-GSK3β (Ser9) and β-catenin in SH-SY5Y cells. Pretreatment with PAE for 2 h suppressed the decrease in expression of above proteins trigged by MPP^+^, and the higher the concentration, the more significant the effect (Fig. 6A–D). To confirm the effect of PAE on AKT/ GSK3β/β-catenin pathway, MK-2206 (2 μM), an inhibitor of AKT, was added to the wells. As shown in Fig. 6E–H, PAE-induced phosphorylation of AKT, GSK3β (Ser9) as well as the increased expression of β-catenin was blocked by MK-2206. Taken together, these results suggest that PAE treatment can result in the activation of AKT/ GSK3β/β-catenin pathway in SH-SY5Y cells.

### AKT pathway activation was essential for the attenuation of mitochondrial dysfuntion and ER stress induced by PAE in SH-SY5Y cells

**Fig. 7:**
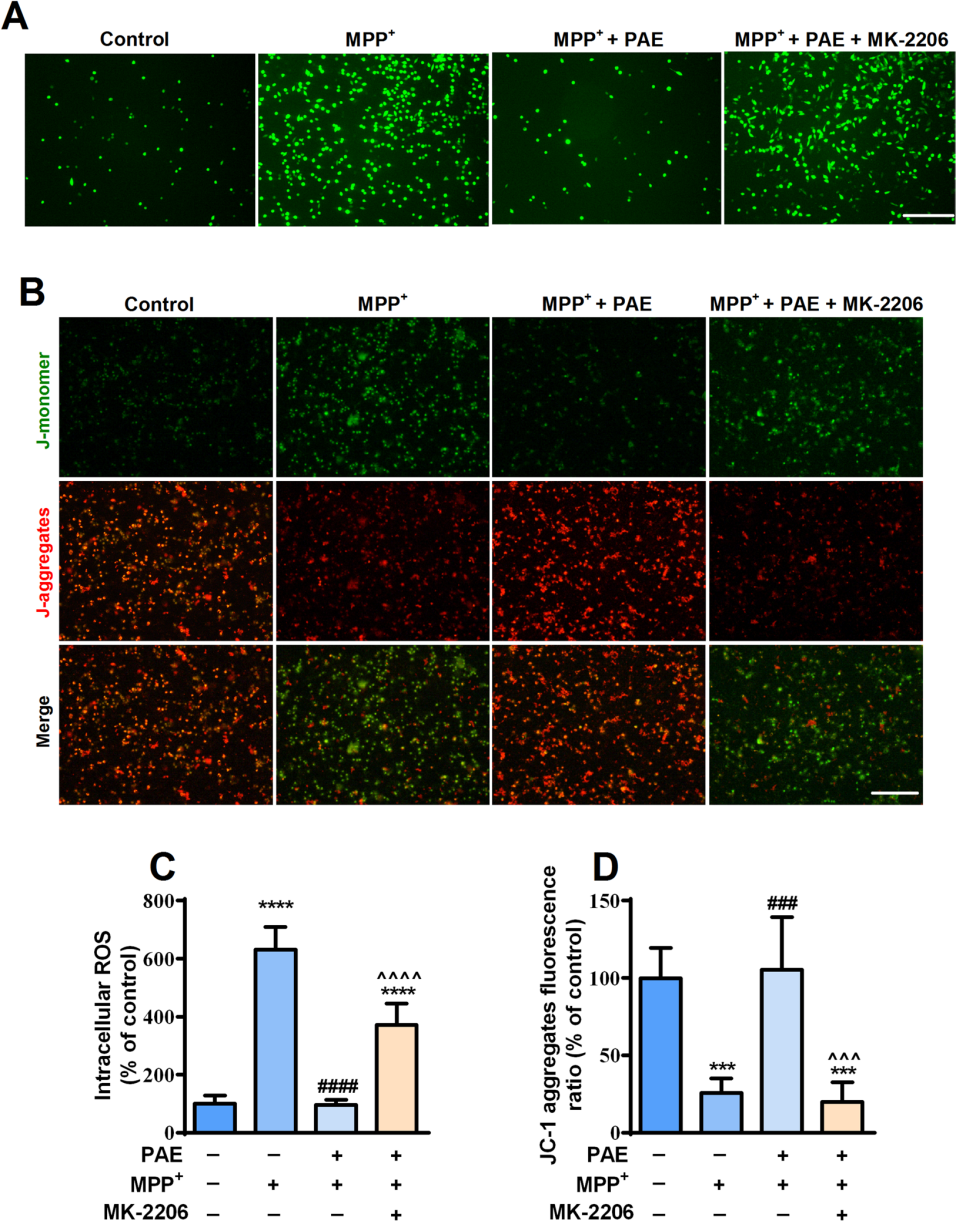
PAE inhibits ROS accumulation and restores Δψm through AKT pathway activation. **A** DCFH-DA assay was performed to detect the intracellular ROS level in each group, bar = 200 μm. **B** JC-1staining was performed to evaluate the Δψm in each group, bar = 200 μm. **C** The inhibition of PAE on ROS overproduction was attenuated by MK-2206. **D** PAE suppressed the reduction of JC-1 aggregates ratio caused by MPP^+^, while MK-2206 blocked the effect of PAE. Results are presented as mean ± SD, n = 3, ^***^p < 0.001, ^****^p < 0.0001, *vs.* control group, ^###^*p* < 0.001, ^####^*p* < 0.0001, *vs.* MPP^+^ treated group, ^^^^^*p* < 0.001,^^^^^^*p* < 0.0001, *vs.* MPP^+^ + PAE group

**Fig. 8:**
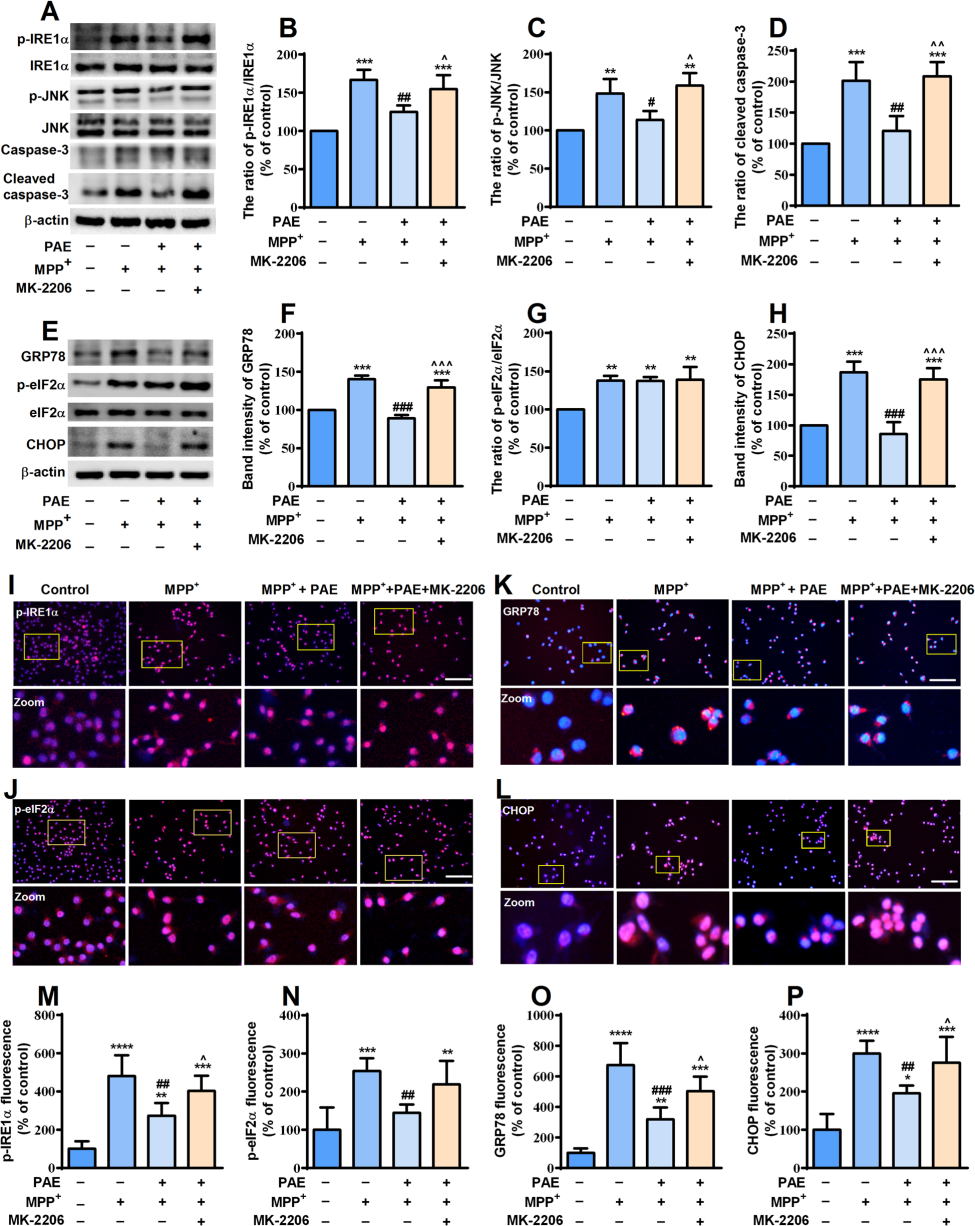
PAE attenuates ER stress through AKT pathway activation. (**A**, **E**) The expression of ER stress indicators in each group was determined by western-blot. PAE pretreatment reserved the up-regulation of p-IRE1α/IRE1α ratio (**B**), p-JNK/JNK ratio (**C**), cleaved caspase-3 (**D**), GRP78 (**F**) and CHOP (**H**) caused by MPP^+^, whereas MK-2206 attenuated the effect of PAE on above proteins (n = 3). The expression of p-IRE1α (**I**), p-eIF2α (**J**), GRP78 (**K**) and CHOP (**L**) was then examined by immunofluorescence staining (bar = 100 μm). MK-2206 blocked the effects of PAE on p-IRE1α (**M**), p-eIF2α (**N**), GRP78 (**O**) and CHOP (**P**), n = 3. Data are presented as mean ± SD, ^*^p < 0.05, ^**^p < 0.01, ^***^p < 0.001, ^****^p < 0.0001, *vs.* control group, ^#^*p* < 0.05, ^##^*p* < 0.01, ^###^*p* < 0.001, *vs.* MPP^+^ treated group, ^^^*p* < 0.05, ^^^^*p* < 0.01, ^^^^^*p* < 0.001, *vs.* MPP^+^ + PAE group

To investigate the role of AKT pathway in the attenuation of mitochondrial dysfuntion and ER stress mediated by PAE, we detected the level of intracellular ROS, the change of Δψm and the expression of proteins related to ER stress after the application of MK-2206. SH-SY5Y cells were pretreated with or without MK-2206 (2 μM) in the presence or absence of PAE (1.6 mg/mL) and then exposed to MPP^+^ for 48 h, ROS and Δψm were determined by DCFH-DA and JC-1 staining, respectively. Consistent with the results shown in Fig. 3, MPP^+^ exposure promoted ROS accumulation and reduced Δψm, pretreatment with PAE (1.6 mg/mL) inhibited the effect of MPP^+^. Interestingly, treating the cells with MK-2206 markedly blocked the effects of PAE on ROS and Δψm (Fig. 7). The expression of ER stress-related key proteins was examined by western blot and immunostaining. As shown in Fig. 8A–H, PAE (1.6 mg/mL) suppressed the up-regulation of p-IRE1α, p-JNK, GRP78, p-eIF2α, CHOP as well as cleaved caspase-3 induced by MPP^+^, while MK-2206 blocked the effects of PAE on above proteins. As expected, the immunostaining results were consistent with western blot that the inhibiting effect of PAE on ER stress-related proteins was attenuated by MK-2206 (Fig. 8I–P). These results indicate that PAE suppress the mitochondrial dysfuntion and ER stress caused by MPP^+^ through activation of AKT pathway.

### PAE improved the behavioral outcomes of MPTP-treated mice through AKT pathway

**Fig. 9:**
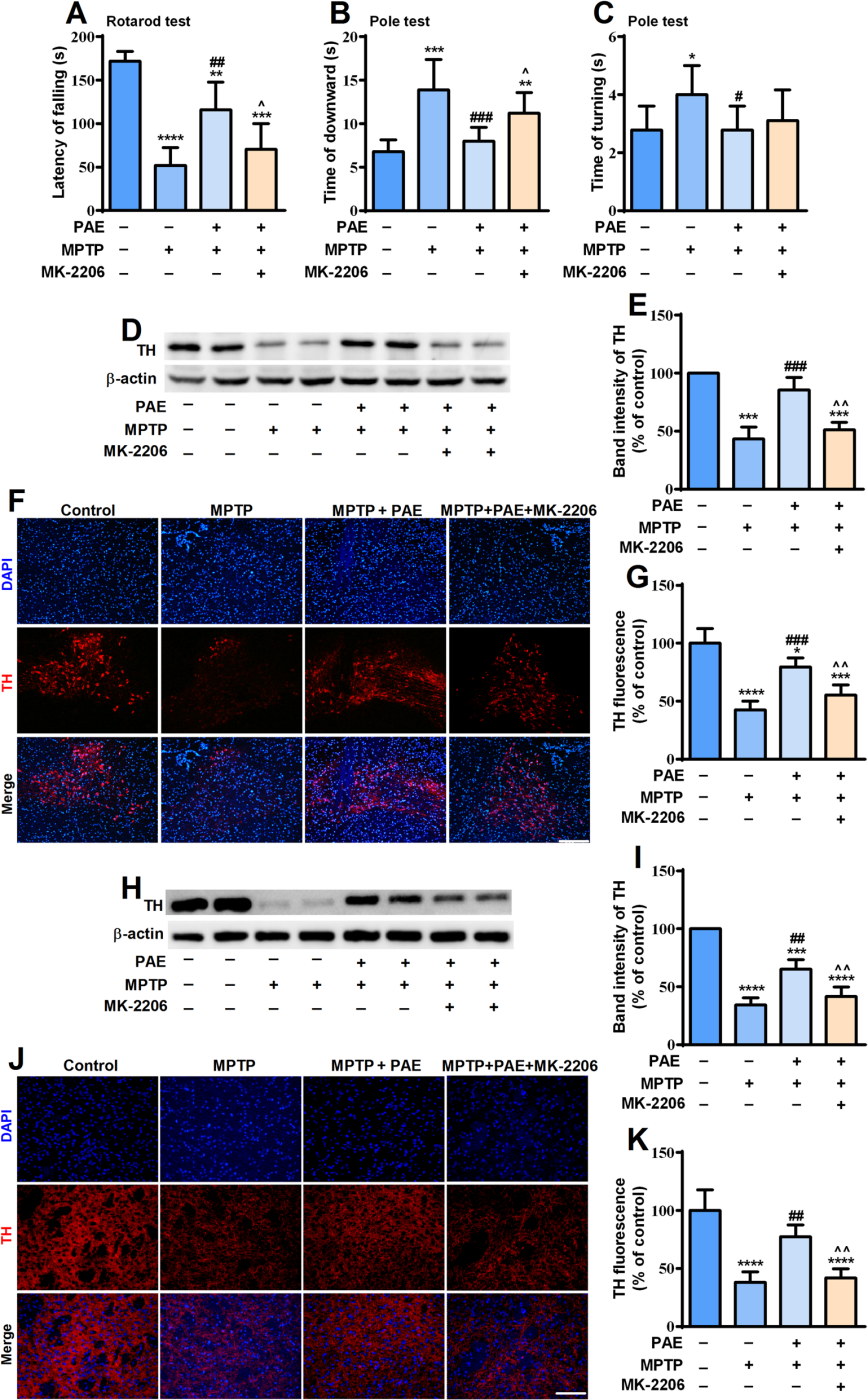
PAE improves the behavioral deficits and inhibits the loss of dopaminergic neurons in substantia nigra and striatum of MPTP-treated mice through AKT pathway. **A** The performance of mice in Rotarod test. PAE administration increased the time of MPTP-treated mice staying on the rod, while MK-2206 blocked the effect of PAE (n = 8). **B** The graph showed the time that the mice took to climb down the pole. PAE reduced the time of MPTP-treated mice took to climb down the pole, MK-2206 attenuated the effect of PAE (n = 8). **C** The graph showed the time that the mice took to turn around in the pole test (n = 8). The representative western-blot analysis of TH expression in the substantia nigra (**D**) and striatum (**H**). PAE treatment inhibited the down-regulation of TH in substantia nigra and striatum caused by MPTP, MK-2206 blocked the effect of PAE (**E**, **I**). PAE prevented the loss of TH-positive neurons in the substantia nigra (**F**, **G**) and TH-positive fiber density in striatum (**J**, **K**) determined by immunofluorescence staining, MK-2206 blocked the effect of PAE, bar = 200 μm, n = 3. Data are presented as mean ± SD, ^*^p < 0.05, ^**^p < 0.01, ^***^p < 0.001, ^****^p < 0.0001, *vs.* control group, ^#^*p* < 0.05, ^##^*p* < 0.01, ^###^*p* < 0.001, *vs.* MPTP group, ^^^*p* < 0.05, ^^^^*p* < 0.01, *vs.* MPTP + PAE group

In order to examine the neuroprotective effects of PAE in vivo, intraperitoneal injection of MPTP was used to induce PD-like symptoms in mice. PAE and MK-2206 were administrated according to the schedule described in “Materials and methods” section. The rotarod test and pole test are commonly used to evaluate the coordination ability of mice [33]. In the rotarod test, MPTP-treated mice maintained a significant shorter time on the rod than control mice, while PAE administration markedly prolonged the residing time of MPTP-treated mice (Fig. 9A). In the pole test, the time spent to completely turn downward (T-turn) and the time spent to reach the bottom of rod (T-down) were measured. The mice in the MPTP-treated group had a much longer T-turn and T-down than the vehicle group, while PAE treatment significantly reduced the T-turn and T-down of MPTP-treated mice (Fig. 9B, C). These data indicated that PAE could ameliorate motor deficits in MPTP-treated mice. To investigate whether AKT pathway was involved in behavioral improvement triggered by PAE, an AKT inhibitor, MK-2206 (100 mg/kg, i.g.) was given every other day starting from day 3, for a total of six times.. As shown in Fig. 9A–C, MK-2206 partially blocked the improving effects of PAE on motor deficits caused by MPTP. Taken together, these results suggest that PAE can improve the behavioral outcomes of MPTP-treated mice partly through AKT pathway.

### PAE prevented the loss of dopaminergic neurons in the substantia nigra and striatum of MPTP-treated mice through AKT pathway

The degeneration of dopaminergic neurons in the substantia nigra pars compacta (SNpc) and the striatum is a typical pathological feature of PD. We then investigated whether PAE could prevent the loss of dopaminergic neurons in the SNpc and striatum. Immunofluorescent staining showed that MPTP led to a significant loss of TH-positive neurons in SNpc and reduced TH fiber density in striatum of mice, while PAE treatment significantly increased the number of TH-positive neurons in aforementioned brain regions (Fig. 9). The results of western blot showed that PAE reversed the down-regulation of TH caused by MPTP in both SNpc and striatum (Fig. 9), which were consistent with results of immunofluorescent staining. However, MK-2206 treatment blocked the effects of PAE, indicating by the reduced number of TH-positive neurons as well as the decreased expression of TH in SNpc and striatum. These data imply that PAE can prevent the loss of dopaminergic neurons in the SNpc and striatum of PD model mice, and AKT pathway is involved in the neuroprotective effects of PAE.

### PAE activated AKT pathway and alleviated ER stress in the substantia nigra and striatum of MPTP-treated mice

**Fig. 10:**
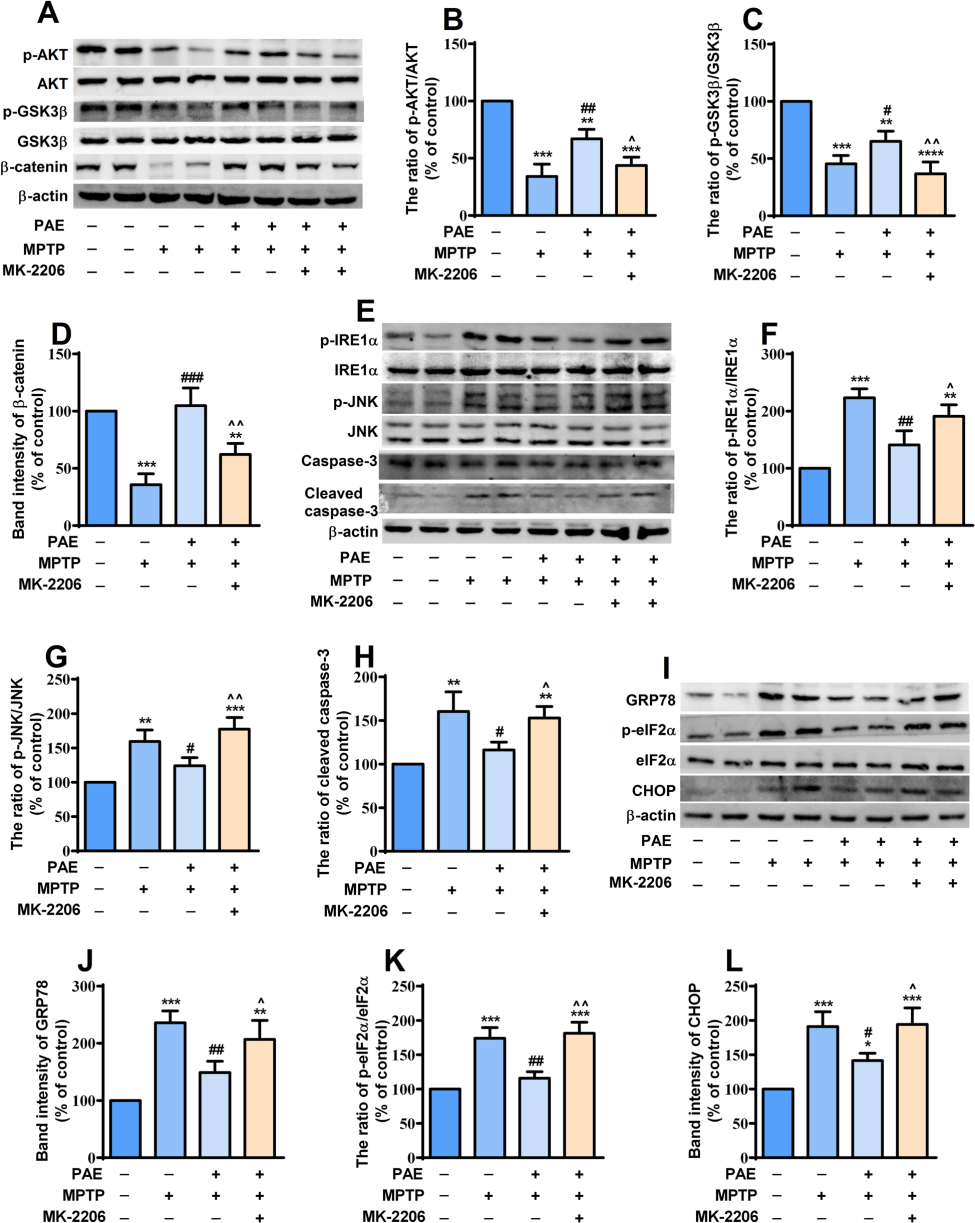
PAE attenuates the ER stress in the substantia nigra of MPTP-treated mice via AKT pathway. **A** The expression of AKT-pathway related molecules in the substantia nigra was detected by western-bot. PAE administration attenuated the reduction of p-AKT/AKT ratio (**B**), p-GSK3β/ GSK3β (**C**) and β-catenin (**D**). **E**, **I** The expression of indicators related to ER stress in the substantia nigra was examined. PAE suppressed the up-regulation of p-IRE1α/IRE1α ratio (**F**), p-JNK/JNK (**G**), cleaved caspase-3 (**H**), GRP78 (**J**), p-eIF2α/ eIF2α ratio (**K**) and CHOP (**L**) in the substantia nigra of MPTP-treated mice, MK-2206 blocked the effects of PAE. Data are presented as mean ± SD, n = 3, ^*^p < 0.05, ^**^p < 0.01, ^***^p < 0.001, *vs.* control group, ^#^*p* < 0.05, ^##^*p* < 0.01, ^###^*p* < 0.001, *vs.* MPTP group, ^^^*p* < 0.05, ^^^^*p* < 0.01, *vs.* MPTP + PAE group

**Fig. 11:**
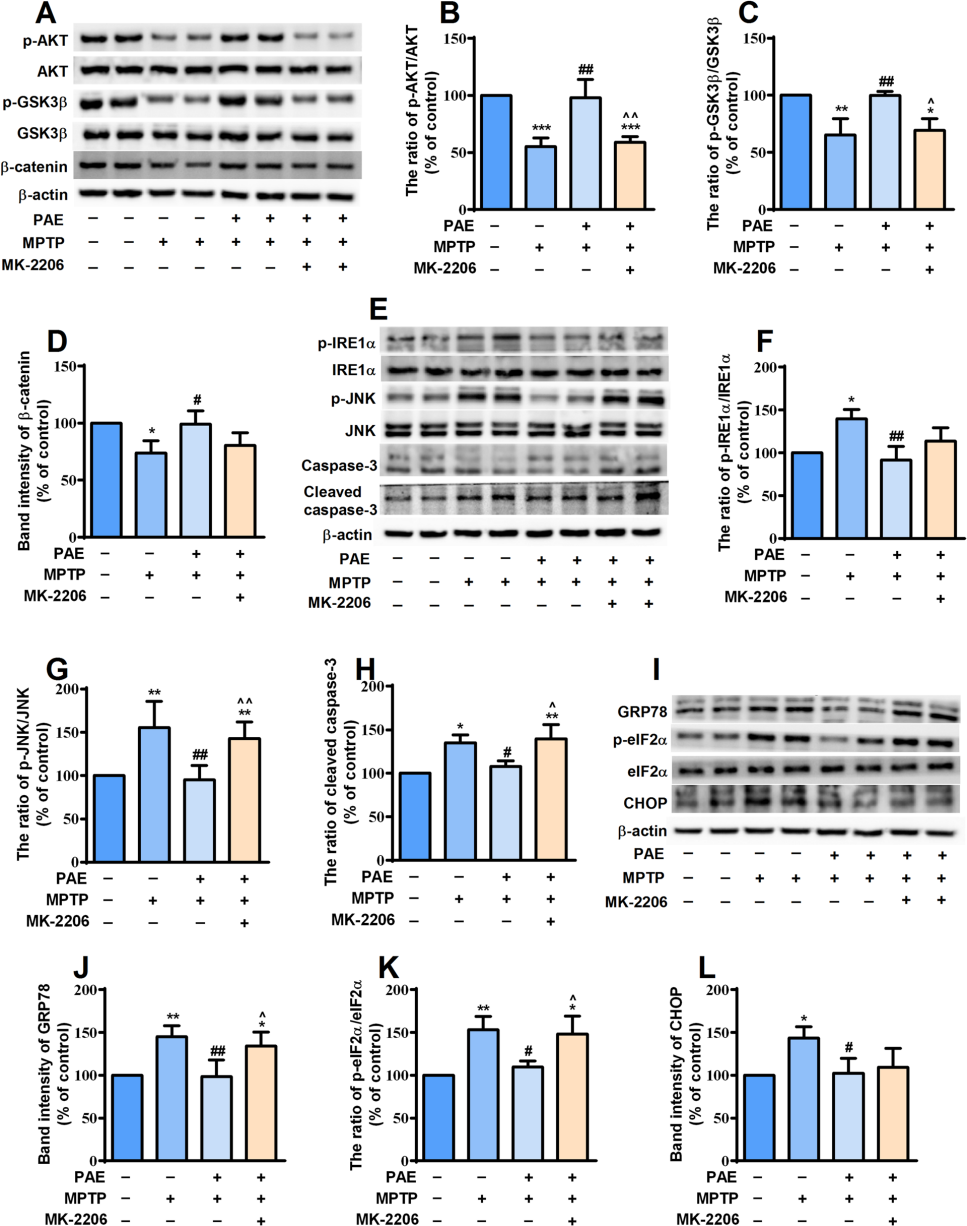
PAE attenuates the ER stress in the striatum of MPTP-treated mice via AKT pathway. **A** The expression of AKT-pathway molecules in the striatum was detected by western-bot. PAE administration attenuated the reduction of p-AKT/AKT ratio (**B**), p-GSK3β/ GSK3β (**C**) and β-catenin (**D**). **E**, **I** The expression of indicators related to ER stress in the striatum was examined. PAE suppressed the up-regulation of p-IRE1α/IRE1α ratio (**F**), p-JNK/JNK (**G**), cleaved caspase-3 (**H**), GRP78 (**J**), p-eIF2α/ eIF2α ratio (**K**) and CHOP (**L**) in the striatum of MPTP-treated mice, MK-2206 blocked the effects of PAE. Data are presented as mean ± SD, n = 3, ^*^p < 0.05, ^**^p < 0.01, ^***^p < 0.001, *vs.* control group, ^#^*p* < 0.05, ^##^*p* < 0.01, ^###^*p* < 0.001, *vs.* MPTP group, ^^^*p* < 0.05, ^^^^*p* < 0.01, *vs.* MPTP + PAE group

We then determined if there were any alterations in the AKT signaling pathway in the SNpc and striatum of MPTP-treated mice. The results showed that the expression levels of p-AKT, p-GSK3β and β-catenin significantly decreased in both SNpc and striatum of MPTP-treated mice. PAE treatment reversed the down-regulation of these proteins, while MK-2206 suppressed the effects of PAE (Figs. 10A–D, 11A–D). These data suggested that PAE could activate AKT /GSK3β /β-catenin pathway in the SNpc and striatum of PD model mice. Having established that PAE exerted neuroprotective effects through attenuating ER stress in in vitro experiments, we asked whether PAE had any effect on ER homeostasis in the brains of MPTP-insulted mice. Western blot analysis was performed to detect the expression of key molecules related to ER stress. The results showed that the expression of p-IRE1α, p-JNK, GRP78, p-eIF2α, CHOP as well as cleaved caspase-3 increased significantly in both SNpc (Fig. 10E–L) and striatum (Fig. 11E–L) of PD model mice, implying that the level of ER stress was enhanced and apoptosis was induced in the brains of MPTP-challenged mice. However, ER stress was attenuated in the SNpc and striatum of mice treated with PAE, indicating by the down-regulation of the above proteins compared with MPTP-treated mice. The alleviating effect of PAE on ER stress was inhibited by MK-2206 (Figs. 10, 11). These data suggest that PAE ameliorates ER stress in the SNpc and striatum of PD model mice probably through activating AKT pathway.

## Discussion

As a prevalent neurodegenerative disease, PD remains a great challenge to the public health, especially the elderly. However, the currently available drugs cannot slow down the progression of PD and frequently produce unbearable side effects. Traditional Chinese medicines (TCM) may provide an option for the treatment of PD due to their multi-targeting properties. In the current study, the neuroprotective activity of ethanol extract of *P. americana* (PAE) against PD was evaluated using both in vitro and in vivo models. MPP^+^ treatment significantly reduced the cell viability, promoted intracellular ROS accumulation, decreased mitochondrial membrane potential, enhanced ER stress and induced evident cell apoptosis in SH-SY5Y cells. However, PAE pretreatment significantly reversed the aforementioned changes caused by MPP^+^. We then demonstrated that TMAO (a ROS inducer) or tunicaymycin (an ER stress inducer) both suppressed the protective effects of PAE. Furthermore, we found that the inhibitory effect of PAE on ER stress was probably mediated by AKT/GSK-3β/β-catenin pathway. These data suggest that PAE protects neuronal cells against MPP^+^-induced toxicity through inhibiting ER stress via AKT-dependent pathway. In MPTP-induced mice model of PD, PAE treatment improved the motor deficits and suppressed dopaminergic neurons loss in the SNpc and striatum. Moreover, PAE blocked the activation of pathways controlling ER stress in these two brain regions. Nevertheless, the above changes exerted by PAE were markedly suppressed by treatment with AKT inhibitor MK-2206. To our knowledge, this is the first study investigating the effects of *P. americana* on PD and showing that PAE can exert neuroprotective activities against PD in both in vitro and in vivo models.

The diversity of components endows the TCM with multiple pharmacological activities. *P. americana* contains numerous bioactive components, for example, studies have shown that the presence of phenolic derivatives, dopamine derivatives, coumarin and cyclic dipeptides confers it with various biological function including anti-inflammatory, analgesic, antimicrobial and anticancer activities[41, 42]. In the present study, the results of LC–MS/MS analysis also showed that the major components of PAE were amino acids, neurotransmitters and their derivatives (data can be found in our previous report) [22]. These compounds may be the key bioactive substances of PAE that can exert neuroprotective effects against PD.

MPP^+^ is a toxic compound with similar structure to dopamine, so it can be easily transported into cells via the dopamine transporters. SH-SY5Y cell line possesses numerous neuronal properties and a catecholaminergic phenotype of dopaminergic neuron, including the expression of tyrosine hydroxylase, dopamine-β-hydroxylase, and dopamine transporters [31]. Exposing SH-SY5Y cells to MPP^+^ has been widely used as an in vitro experimental model for PD research [25, 26]. However, it should be noted that differentiated and undifferentiated cells have different tolerance to MPP^+^. In dopamine neurons, MPP^+^ exerts neurotoxic effects by inhibiting the activity of mitochondria respiratory chain complex I, then reducing ATP production and producing a large amount of ROS [43]. ROS not only leads to the loss of mitochondrial membrane potential and causes mitochondrial dysfunction, but also strongly induces ER stress. ROS accumulation can destroy the redox homeostasis of ER and result in the aggregation of oxidized proteins, which aggravates the unfolding or misfolding of proteins and cause ER stress [44]. Mitochondrial dysfunction and ER stress has been recognized as important contributors to dopaminergic neuron loss in the process of PD [45–48]. Therefore, whether PAE could prevent MPP^+^-induced dopaminergic neurons loss was examined firstly in this study. Pretreatment with PAE increased the cell viability and inhibited cell apoptosis caused by MPP^+^ in a concentration-dependent manner. Activated caspase-3 is the executor of apoptosis, MPP^+^ was found to increase the expression of cleaved caspase-3 and pro-apoptosis protein Bax, while reducing the level of anti-apoptosis protein Bcl-2. The effect of MPP^+^ was attenuated by PAE treatment. These results suggest that PAE can protect SH-SY5Y cells against MPP^+^-induced cell loss. To explore whether the neuroprotective effects of PAE were mediated by inhibiting mitochondrial dysfunction and ER stress, we detected ROS, Δψm and the key indicators of ER stress. Under ER stress, GRP78 was disassociated from ER stress transducers, including PERK and IRE1α, and then the UPR was initiated. Under prolonged or excessive stress, IRE1α can promote cell death by activating ASK1, followed by the activation of JNK and caspase-3 [15]. PERK is a type I ER transmembrane protein containing a cytosolic kinase domain [49]. PERK activation phosphorylates the residue serine 51 of α subunit of eukaryotic translation initiation factor (eIF2α) [50], then leading to the reduced protein synthesis and the promotion of ATF4 translation [51]. ATF4 in turn upregulates the transcription of the gene encoding pro-apoptotic factor CCAAT-enhancer-binding protein homologous protein (CHOP), which further activates the caspase-3 apoptotic cascade and promotes apoptosis [52]. In this study, PAE suppressed the accumulation of ROS, the loss of Δψm as well as the activation of IRE1α and PERK pathways caused by MPP^+^. Furthermore, we demonstrated that the inhibiting effects of PAE against MPP^+^-induced cell loss were abolished by TMAO (a ROS inducer) or tunicaymycin (an ER stress inducer), whereas NAC (a free radical scavenger) or 4-PBA (an ER stress inhibitor) exhibited similar protective activities to that of PAE. Our results indicate that PAE may exert neuroprotective effect through suppressing the mitochondrial dysfunction and ER stress.

1-Methyl-4-phenyl-1,2,3,6-tetrahydropyridine (MPTP) is the precursor of MPP^+^ and an environmental neurotoxin widely used to establish animal models of PD, as it selectively destroys dopaminergic neurons and induces symptoms of PD. To further confirm the neuroprotective activity of PAE, we investigated its effects in an in vivo PD mouse model induced by MPTP. The rotarod test and pole test are important methods for detecting the motor deficits of PD animals by evaluating coordination and balancing abilities [33]. PAE treatment increased the time of MPTP-treated mice retaining on the rotarod, and reduced the time taken to turn around and downward in pole test. It indicates that PAE can improve the behavioral dysfunction of PD mice. Tyrosine hydroxylase (TH) is the rate-limiting enzyme in dopamine synthesis, and its deficiency is believed to contribute to dopaminergic insufficiency and trigger the onset of PD [53]. Our study revealed that PAE significantly prevented the loss of TH-positive cells in the SNpc and striatum of MPTP-treated mice. The result of western-bolt also found that TH expression was reduced in both regions, which was consistent with previous studies that showed the reduced TH expression correlated with behavioral deficits in toxin-induced PD animal model [54, 55]. We also saw that PAE suppressed the activation of IRE1α and PERK pathways as well as the up-regulation of cleaved caspase-3, indicating that PAE could attenuate ER stress and inhibit the subsequent cell apoptosis caused by MPTP. These data suggest that PAE may prevent dopaminergic neuron loss through inhibiting ER stress, which is consistent with the findings in in vitro experiments.

AKT signaling pathway plays a crucial role in maintaining neuronal survival against various apoptotic stimuli. The phosphorylation of AKT can attenuate neuronal death under various harmful conditions [56, 57], whereas inhibition of AKT phosphorylation promotes neuronal injury [58]. AKT has been considered as a plausible therapeutic target in PD [57]. In the present study, we found that the phosphorylation of AKT decreased significantly after exposing the cells to MPP^+^, while PAE markedly enhanced p-AKT level in a concentration-dependent manner. GSK3β is a downstream target of AKT, and AKT activation can promote the phosphorylation of GSK3β at serine-9 and then induce the inhibition of GSK3β activity, consequently exerting neuroprotective effects [59, 60]. Our data showed that PAE treatment reversed the reduction of p-GSK3β at serine-9 caused by MPP^+^, indicating that PAE could inhibit GSK3β activity. As a substrate of GSK3β, β-catenin is a key mediator of cell survival [40]. β-catenin may facilitate cell survival via enhancing anti-oxidant activity and preventing intracellular ROS accumulation [61], thereby maintaining the homeostasis of mitochondria and inhibiting the activation of related apoptotic pathways [62]. GSK3β promotes the proteasomal degradation of β-catenin and then causes cell apoptosis, whereas GSK3β inhibition up-regulates the expression of β-catenin and exerts neuroprotective effects in models of PD [63–65]. Consistently, we also found that the down-regulation of β-catenin caused by MPP^+^ was attenuated by PAE treatment. Hence, we propose that AKT/GSK3β/β-catenin pathway is involved in the protective effects of PAE. To further clarify the role of AKT-dependent pathway, MK-2206 (an AKT inhibitor) was applied. We found that MK-2206 blocked the effects of PAE on GSK3β and β-catenin in in vitro experiments. Similar results were also seen in the SNpc and striatum of mice treated with MK-2206 in in vivo study. These results indicate that AKT/GSK3β/β-catenin pathway is involved in the effects of PAE. As mentioned above, β-catenin activation could attenuate cell damage through enhancing anti-oxidant activity and inhibiting oxidative stress (ROS accumulation). ROS is a strong inducer of ER stress. To testify whether the inhibiting effects of PAE on ROS production and ER stress were mediated by AKT-dependent pathway, the intracellular ROS level, Δψm and expression of key molecules related with ER stress were detected after MK-2206 application. We found that MK-2206 abolished the inhibitory effects of PAE on ROS accumulation, the loss of Δψm as well as the activation of IRE1α and PERK pathways in the cultured SH-SY5Y cells. Similarly, the attenuation of ER stress exerted by PAE was also blocked by MK-2206 in the SNpc and striatum of MPTP-treated mice. These data suggest that PAE suppress ROS overproduction and ER stress through activating AKT-dependent pathway.

## Conclusion

**Fig. 12:**
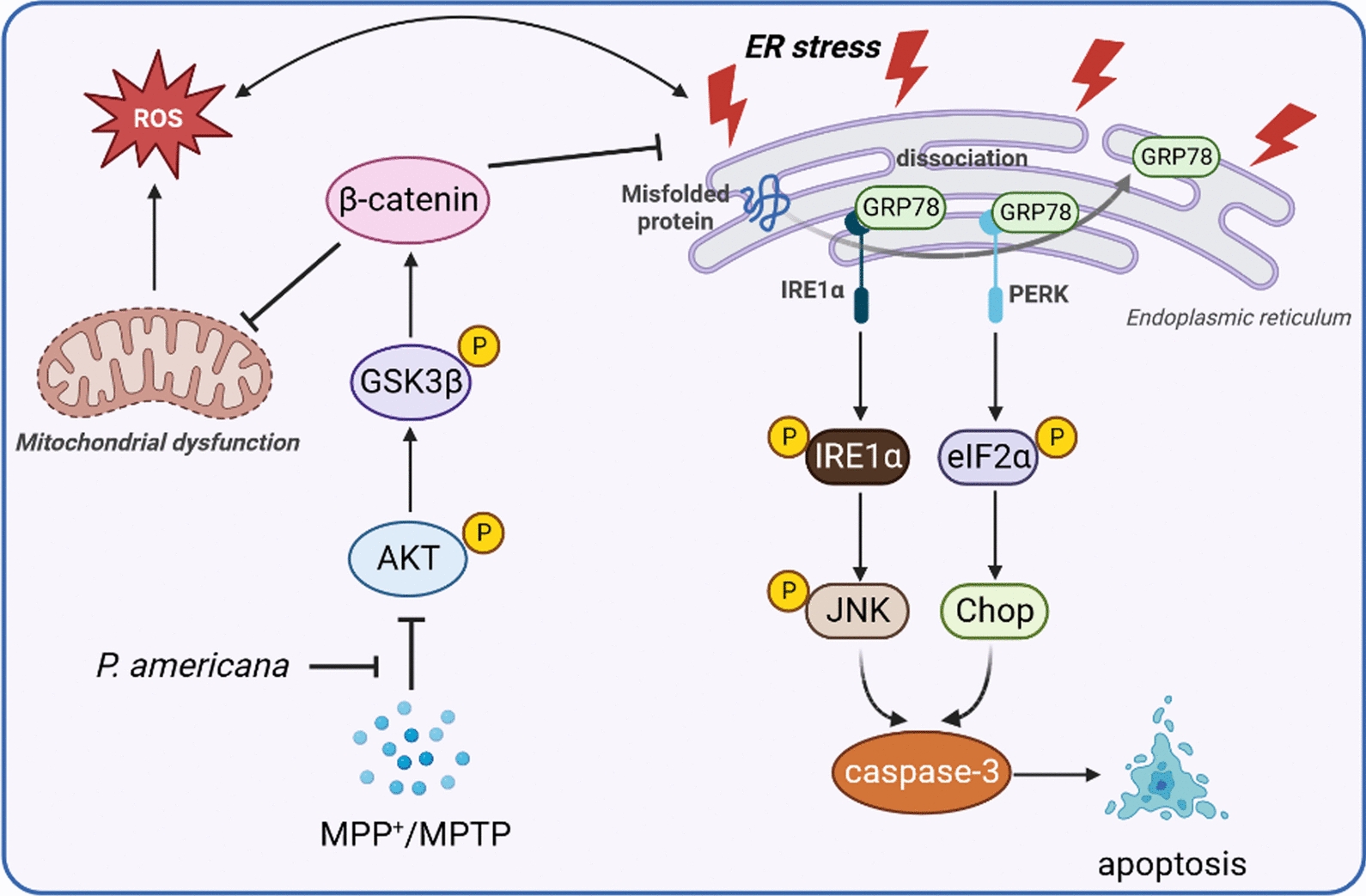
Schematic diagram of the neuroprotective effects of PAE in experimental models of Parkinson’s disease by inhibiting ER stress via AKT-dependent pathway

In summary, the present study provides evidence that the ethanol extract of *P. americana* can efficiently inhibit MPP^+^-induced cell death in SH-SY5Y cells as well as ameliorate motor deficits and prevent dopaminergic neurons loss in MPTP-induced PD model mice. Mechanism study reveals that *P. americana* exerts neuroprotective effects probably through inhibiting ROS-mediated ER stress in AKT-dependent pathway (Fig. 12). This is the first study to investigate the neuroprotective role of *P. americana* in in vitro and in vivo PD models and also address the possible underlying mechanisms. The therapeutic potential of *P. americana* examined in our study indicates that it is a promising source for developing new drugs against PD. However, this study also has some limitations and they may be the potential directions of future research. First, the PAE used in this study is a mixture, it is still unclear which substance(s) play the main role in its protective effects, so the bioactive components present in the *P. americana* are still need to be further separated and identified for their neuroprotective efficacy. Second, in this study, we only assessed the neuroprotective effects of PAE on one type of PD animal model (induced by MPTP), it is unclear whether the results are universally applicable or not. The protective effect of PAE needs to be validated in more types of PD animal models if we want to translate it into clinic in the future.

## Data Availability

The data and materials supporting the current study are available from the corresponding author upon reasonable request.

## Abbreviations

Δψm: Mitochondrial membrane potential
4-PBA: Sodium 4-Phenylbutyrate
ATF6: Activating transcription factor 6
CHOP: C/EBP homologous protein
eIF2α: Eukaryotic initiation factor 2α
ER: Endoplasmic reticulum
GSK3β: Glycogen synthase kinase-3β
GRP78: 78-KDa glucose-regulated protein
IRE1α: Inositol-requiring 1α
JNK: Jun-N-terminal kinase
MPP^+^: 1-Methyl-4-phenyl-pyridinium
MPTP: 1-Methyl-4-phenyl-1, 2, 3, 6-tetrahydro-pyridine
NAC: N-Acetyl-L-cysteine
PD: Parkinson’s disease
PERK: Double-stranded RNA dependent protein kinase-like ER kinase
SNpc: Substantia nigra pars compacta
TH: Tyrosine hydroxylase
TMAO: Trimethylamine N-Oxide
UPR: Unfolded protein response
